# House Governor or Medical Superintendent? I

**Published:** 1892-08-13

**Authors:** 


### PASSING TOPICS.

### HOUSE GOVERNOR, OR MEDICAL SUPERINTENDENT?—I.

Among the many questions which have been galvanized into fresh life by the issue of the report of the Lords' Select Committee, and the discussion it has given rise to, is the old one, whether it is better that the chief executive authority in a hospital shall be exercised by a lay or a medical officer? Shall control and superintendence be by House Governor or his equivalent, or by Medical Superintendent?

But first, perhaps, it is well to ask whether supreme authority should be vested in any one officer? because at present this is not generally admitted. The Lords' Committee, as we think wisely, appear to have made up their minds that the question should be answered in the affirmative, and in the course of the inquiry searching interrogation was made of many of the secretaries as to the powers they possessed for dealing promptly with certain hypothetical but not improbable circumstances. Some of the witnesses frankly confessed they had no plenary powers, while others had persuaded themselves that they were fully authorized to do anything which appeared to them necessary. But we think it may be taken for granted that except here and there the authority which is direct, personal and palpable, is conspicuously absent, and what we have in its place is a government of somewhat visionary character, whose shadow does duty as a sort of bogey. In their desire to show that something more than this is needed, we find the Lords inclining to an eulogy of the management of the Poor Law Infirmaries, which approaches closely to a despotism.

But authority and despotism are not necessarily allied, and an officer who has been entrusted with supreme power ought not on that account to be the less but rather the more, completely held to his responsibilities. In the case of the voluntary hospitals, however, we have to do with committees, and committees being always sensitively jealous of authority of this description, confer it only rarely, and even then are not always to be depended upon to uphold it ungrudgingly. This reluctance has been well illustrated in the case of the London Hospital, where, rules notwithstanding, the authority of the Governor was admitted to have been allowed to lapse—at the instance of the Committee—in very important particulars. Most committeemen seem to think it is best that the executive authority should be divided among a number of departments, the heads of which are severally independent one of the other, and that the committee should themselves gather up and hold the reins.

This would appear to be a hazardous course, because the authority of a committee is necessarily intermittent and may not be uniform, while what is above all things indispensable to discipline and smooth working is a well founded knowledge, in a hospital no less than in any other organization, that authority is always at hand and ready to be invoked.

Whenever hospital affairs come to be regarded as within the scope of strictly rational treatment, we may be sure that each institution will boast a resident authority capable enough to command respect and strong enough to make itself accepted unquestioningly within its rightful limits. The fact that these limits are imposed rescues authority from a suspicion of despotism and reveals the existence of the higher powers to which it is in its turn subject. Whether this embodiment of authority should be lay or medical in character remains to be considered. Agricola.

